# Community engagement among forest goers in a malaria prophylaxis trial: implementation challenges and implications

**DOI:** 10.1186/s12936-023-04610-6

**Published:** 2023-06-08

**Authors:** Franca Conradis-Jansen, Rupam Tripura, Thomas J. Peto, James J. Callery, Bipin Adhikari, Mom Ean, Monnaphat Jongdeepaisal, Christopher Pell, Panarasri Khonputsa, Riccardo Murgia, Siv Sovannaroth, Olaf Müller, Phaik Yeong Cheah, Arjen M. Dondorp, Lorenz von Seidlein, Richard J. Maude

**Affiliations:** 1 Heidelberg Institute of Global Health, Medical School, Ruprecht-Karls-University Heidelberg, Heidelberg, Germany; 2 Mahidol Oxford Tropical Medicine Research Unit, Faculty of Tropical Medicine, Mahidol University, Bangkok, Thailand; 3 Centre for Tropical Medicine and Global Health, Nuffield Department of Clinical Medicine, University of Oxford, Oxford, UK; 4 Amsterdam Institute for Global Health and Development, Amsterdam, The Netherlands; 5 Amsterdam UMC, Department of Global Health, University of Amsterdam, Amsterdam, The Netherlands; 6 Amsterdam Public Health Research Institute, Global Health Program, Amsterdam, The Netherlands; 7 Center for Parasitology, Entomology and Malaria Control, Phnom Penh, Cambodia; 8 The Open University, Milton Keynes, UK

**Keywords:** Chemoprophylaxis, Clinical trial, Community engagement, Malaria, Southeast Asia

## Abstract

**Background:**

Malaria transmission in Southeast Asia is increasingly confined to forests, where marginalized groups are exposed primarily through their work. Anti-malarial chemoprophylaxis may help to protect these people. This article examines the effectiveness and practical challenges of engaging forest-goers to participate in a randomized controlled clinical trial of anti-malarial chemoprophylaxis with artemether-lumefantrine (AL) versus a control (multivitamin, MV) for malaria in northeast Cambodia.

**Methods:**

The impact of engagement in terms of uptake was assessed as the proportion of people who participated during each stage of the trial: enrolment, compliance with trial procedures, and drug intake. During the trial, staff recorded the details of engagement meetings, including the views and opinions of participants and community representatives, the decision-making processes, and the challenges addressed during implementation.

**Results:**

In total, 1613 participants were assessed for eligibility and 1480 (92%) joined the trial, 1242 (84%) completed the trial and received prophylaxis (AL: 82% vs MV: 86%, p = 0.08); 157 (11%) were lost to follow-up (AL: 11% vs MV: 11%, p = 0.79); and 73 (5%) discontinued the drug (AL-7% vs MV-3%, p = 0.005). The AL arm was associated with discontinuation of the study drug (AL: 48/738, 7% vs 25/742, 3%; p = 0.01). Females (31/345, 9%) were more likely (42/1135, 4%) to discontinue taking drugs at some point in the trial (p = 0.005). Those (45/644, 7%) who had no previous history of malaria infection were more likely to discontinue the study drug than those (28/836, 3%) who had a history of malaria (p = 0.02). Engagement with the trial population was demanding because many types of forest work are illegal; and the involvement of an engagement team consisting of representatives from the local administration, health authorities, community leaders and community health workers played a significant role in building trust. Responsiveness to the needs and concerns of the community promoted acceptability and increased confidence in taking prophylaxis among participants. Recruitment of forest-goer volunteers to peer-supervise drug administration resulted in high compliance with drug intake. The development of locally-appropriate tools and messaging for the different linguistic and low-literacy groups was useful to ensure participants understood and adhered to the trial procedures. It was important to consider forest-goers` habits and social characteristics when planning the various trial activities.

**Conclusions:**

The comprehensive, participatory engagement strategy mobilized a wide range of stakeholders including study participants, helped build trust, and overcame potential ethical and practical challenges. This locally-adapted approach was highly effective as evidenced by high levels of trial enrolment, compliance with trial procedures and drug intake.

## Background

In the Greater Mekong Subregion (GMS), malaria incidence has greatly decreased in the past decade [1] and transmission is increasingly confined to populations in remote, forested areas [2]. To achieve malaria elimination, several strategies have been deployed, such as active case detection (ACD), focused screening and treatment (FSAT) by rapid diagnostic test (RDT), and reactive case detection (RACD) to specifically target positive malaria cases identified by village malaria workers (VMWs) through passive case detection and to identify asymptomatic malaria cases by screening household members and neighbouring households with RDTs.

The available, appropriate diagnostic methods are however not sensitive enough to detect low density *Plasmodium* infections [3–5]. Several studies on mass drug administration have shown promising results in trial settings [6–8]. Yet policymakers are hesitant to adopt this aggressive strategy to accelerate malaria elimination. Their reticence is chiefly due to concerns about acceptability, feasibility, and the absence of an “ideal” anti-malarial for this purpose [9].

Vector control strategies (chiefly insecticide-treated bed nets) were not found to be effective against forest-acquired malaria in the GMS [10, 11] because mosquito vectors also bite in the daytime when people are outdoors [12]. In addition, forest goers often shelter in temporary camps where it is difficult to deploy conventional vector control measures. Interrupting transmission in these environments is essential if malaria elimination is to be achieved and new approaches targeted at forest workers must be evaluated. A potential alternative intervention that could protect forest workers from malaria is chemoprophylaxis [13]. Evidence for benefits from this strategy is limited and engaging remote forest workers in research to evaluate this intervention is challenging.

Forest work in Cambodia is often informal, and sometimes illegal, involving logging or poaching in protected areas [14]. Forest goers usually travel in groups with relatives, neighbours or other trusted persons [10]. Because of the illegal nature of many forest activities, their low access to education, and their poor economic status, and the fact that they mainly belong to ethnic groups who speak minority languages, forest goers are marginalized groups [14, 15]. These communities commonly reside in remote villages and are highly mobile due to the nature of their work, sometimes leaving their domicile for weeks without maintaining a fixed abode [14]. Conducting successful research with remote forest going communities presents a range of barriers and challenges [2].

Community engagement is crucial for the implementation of community-based trials in which locally recruited staff play an important role as they are familiar with the local social and cultural context [16, 17]. Building trust and working relationships, perhaps the most important goals of community engagement, can be achieved over time by careful interaction between trial staff and target communities [17]. Practical challenges for collaboration with forest goers include difficult access [10], logistical constraints and the need to work with various stakeholders with differing interests, building trust between marginalized groups, the research team and other stakeholders and identifying the most appropriate methods to explain the aims and activities of the trial in keeping with the background and attitudes of the individuals [17, 18]. Understanding trial community’s views and attitudes is even more important, especially when malaria incidence is declining in the region (e.g. Stung Treng Province). Study communities have questioned how they will benefit from participating in malaria elimination research projects [19]. Responding to their concerns and explaining well the rationale for community-based trials are critical. For instance, in this trial it was essential to explain the implications of forest going patterns, and the need for long-term community (forest-goers) participation [10, 20]. The understanding and perception of the disease, and its risks and implications, are often considered prerequisites for participation and may require appropriate health education [20].

This article describes the community engagement approach for a chemoprophylaxis trial that recruited forest goers in northeastern Cambodia and discusses the main challenges the trial team faced, how they were addressed, and the impact of community engagement on trial participation. The main outcomes of the trial have been reported elsewhere [21].

## Methods

### Trial site and participants

**Fig. 1:**
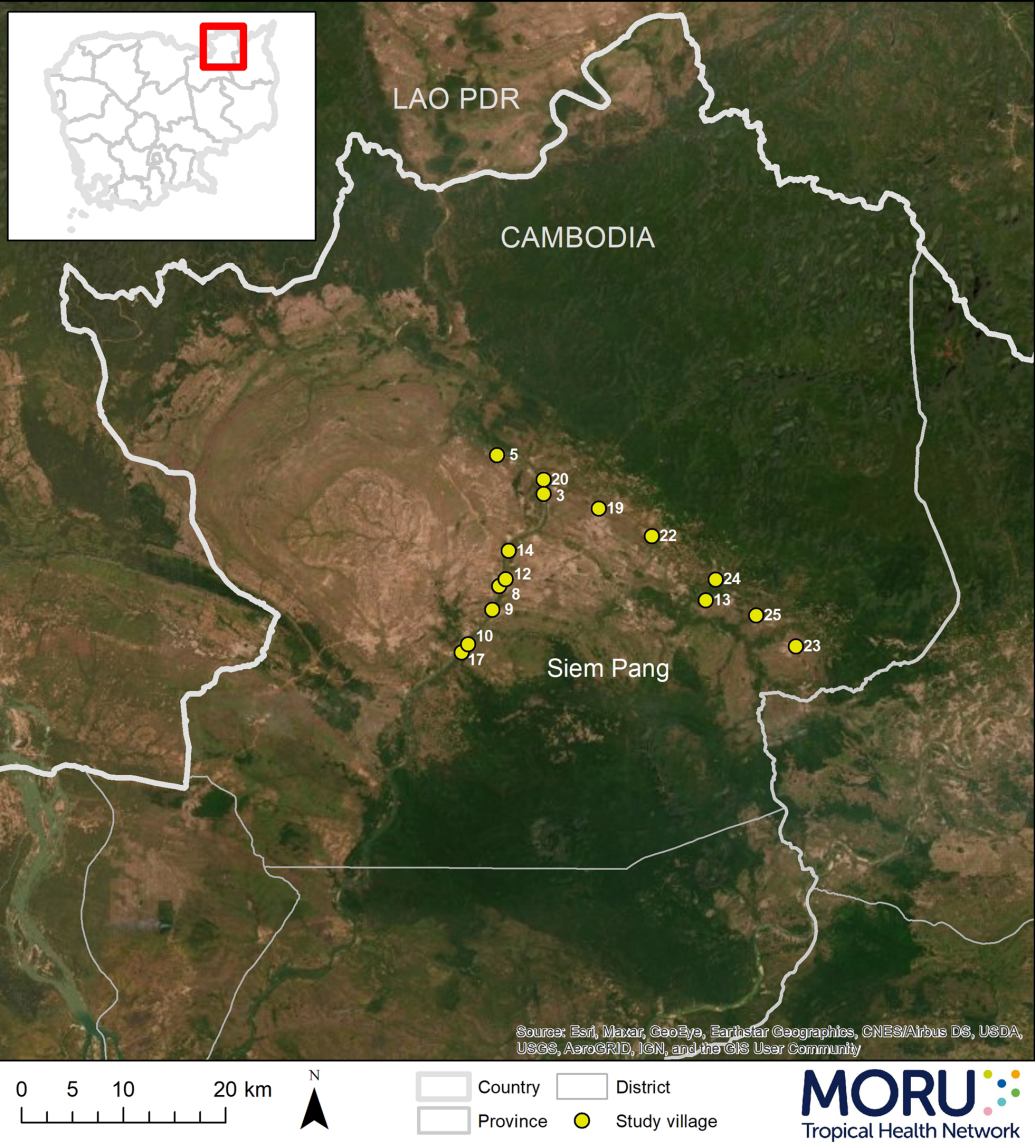
Trial villages

The trial was conducted in Siem Pang District, Stung Treng Province, a remote area in northeast Cambodia, which borders southern Lao PDR (Fig. 1). The estimated population of Siem Pang District was around 26,000 in 2019 [22]. The trial was conducted among forest goers in 15 of 41 villages in the district**.** The villages with highest malaria incidences were selected based on the available data during 2018–19 (source: Malaria Information System, Cambodia https://mis.cnm.gov.kh/). The population of each village in the area varied widely between 98 and 1644 inhabitants. Communities consist of ethnic Khmer along with a large proportion of ethnic minorities, chiefly Kavet, Khe and Lao. Some of the trial villages had the highest malaria incidences among all villages of Siem Pang district during 2018–19 based on the records of the National Center for Parasitology Entomology and Malaria Control, Cambodia (CNM) [23]. Since 2018, the Public Engagement Department of Mahidol Oxford Research Unit (MORU) has been conducting a series of health education programme, such as village drama, traditional songs, arts, comedy theatre, and health talks. Health education topics for this engagement were jointly selected by the community leaders and the engagement team [24].

In 2020, the engagement activities took place in the context of an open-label, individually randomised controlled trial. The trial compared the efficacy of artemether-lumefantrine (AL) as anti-malarial chemoprophylaxis versus a multivitamin (MV), which does not have any anti-malarial properties. AL was provided as 4 tablets twice daily for 3 consecutive days, followed by twice-a-day weekly dosing for a maximum of 4 months. A multivitamin was provided as 1 tablet twice daily for 3 consecutive days and then weekly thereafter for the same duration. The engagement activities began in January 2020, two months prior to the enrollment in the trial, and continued throughout the trial period. Forest goers aged between 16 and 65 years who planned an overnight stay in the forest were invited to participate in the trial. Females with known pregnancy or breastfeeding were excluded. The trial protocol has been published elsewhere [25].

### Community engagement and anticipated challenges

Anti-malarial chemoprophylaxis is a novel approach to prevent and control malaria among targeted populations in the Greater Mekong Subregion (GMS). Engaging community members and motivating apparently healthy forest goers to take prophylactic drugs were crucial to achieve high participation in the trial. From previous research that highlighted perceptions around asymptomatic malaria infections [26–28], it was anticipated that the major challenges for trial implementation would be: (i) to convince healthy individuals to take the study drugs for up to 4 months with monthly follow-up visits; (ii) to reach out effectively to forest goers who are highly mobile and whose work is often illegal [2]; (iii) to ensure all trial participants comprehend the trial rationale and complied with trial procedures despite a range of literacy levels, languages and ethnicities; and (iv) to deal appropriately with perceptions of adverse events and rumours, such as “stealing of blood”, and “infertility” [29].

### Engagement strategies and activities

**Fig. 2:**
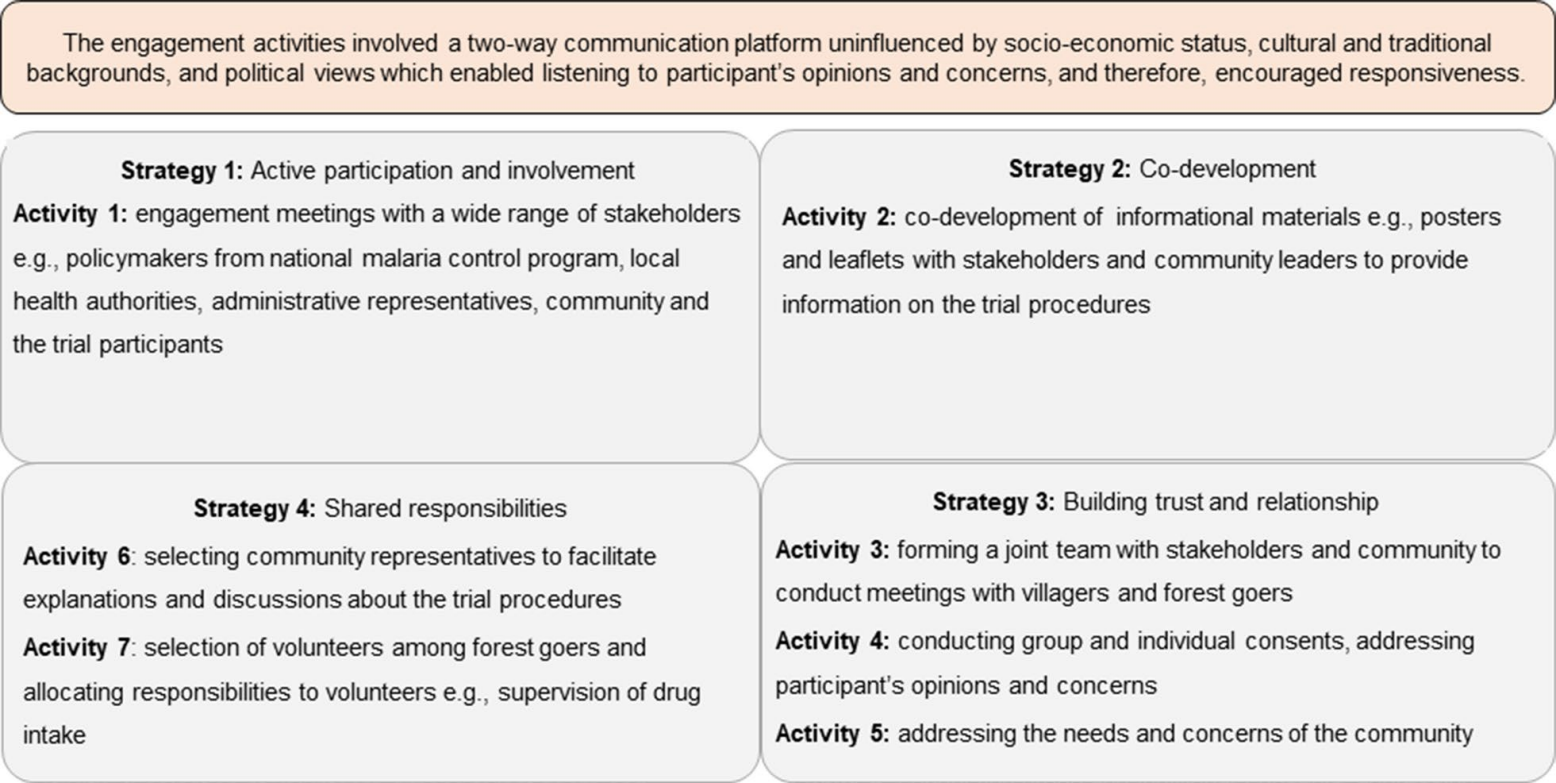
Key engagement strategies and activities

Community engagement strategies were adopted to engage community members and forest goers to ensure that they understood the trial procedures and potential benefits and harms associated with the trial drugs, and to make independent judgements and decisions about whether to participate in the trial without any external influence. To achieve this, we enlisted the following strategies (Fig. 2): active participation and involvement of stakeholders and participants; co-development of trial related informational materials; building trust and relationships; and sharing responsibilities with stakeholders, communities and trial participants. These strategies were partially adapted from earlier studies of mass drug administration conducted on healthy individuals in western Cambodia and Lao PDR [6, 16, 27, 28, 30]. Aligning with the strategies and anticipated challenges, the trial team designed a series of activities, which included: engagement meetings with stakeholders that included policymakers from CNM, health authorities at provincial and district levels, local administrative representatives, the community and trial participants; the co-development of informational materials e.g. posters and leaflets to provide information on the trial; forming a joint team with stakeholders to conduct meetings with community members; conducting group and individual consents; addressing the needs and concerns of the community; selecting community representatives to facilitate explanations and discussions about the trial and procedures; and selecting and allocating responsibilities to forest goer volunteers, e.g. the supervision of drug intake among fellow forest goers. These activities are described in detail below.

1. Engaging stakeholders, communities and participants

**Fig. 3:**
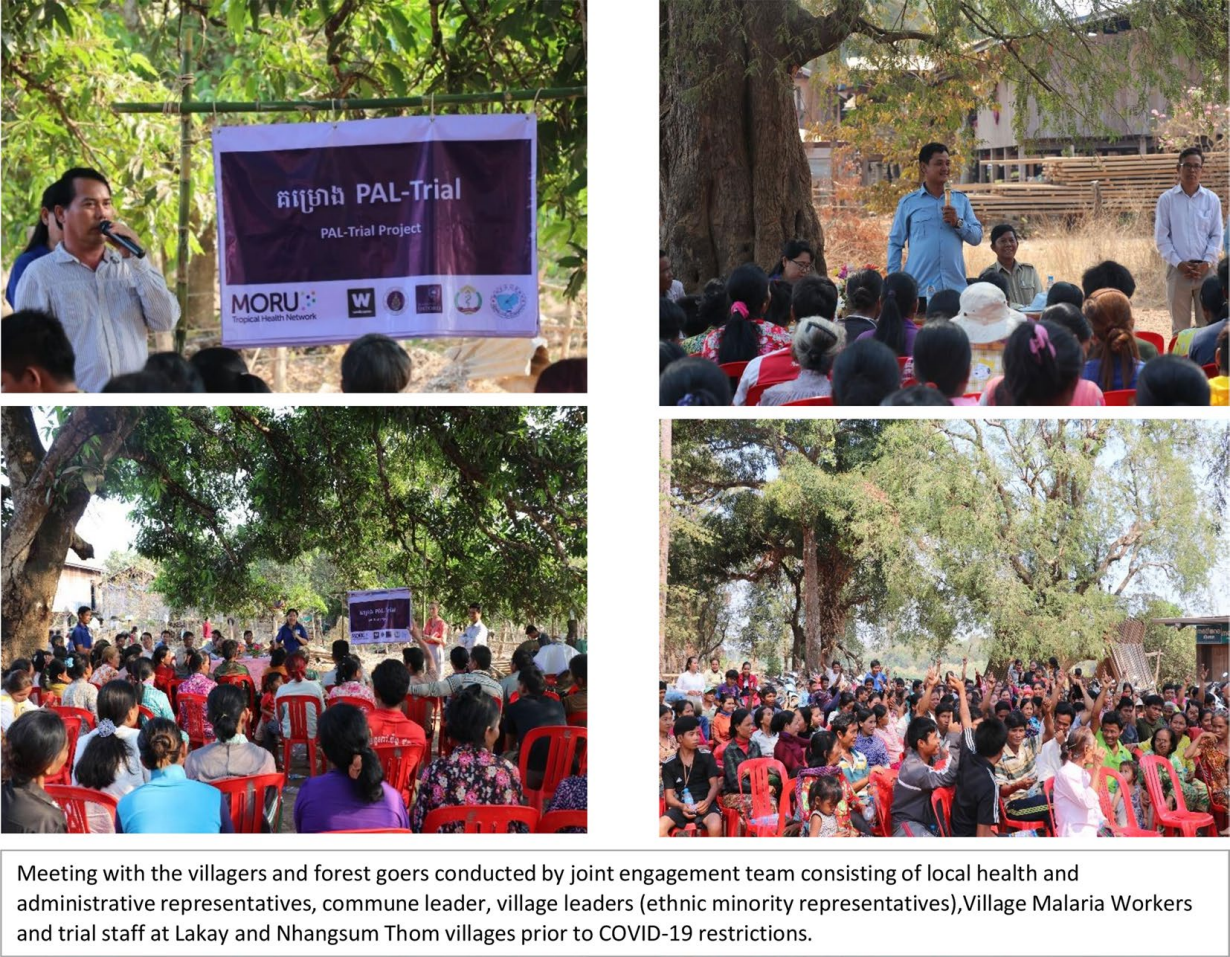
Meetings conducted by joint engagement team

A series of meetings was conducted (small- and large-scale) with national and local health authorities, local administrations, community leaders, health workers and villagers prior to, and during, trial implementation. The engagement meetings were open to all people independent of socioeconomic status, cultural and traditional backgrounds, or political views. The meetings with stakeholders and community members were designed as a two-way communication platform, which enabled listening to participants’ opinions and concerns and encouraged responsiveness. The feedback and recommendations from the meetings were incorporated into the trial protocol, training and study materials that provided information related to the trial and activities. Discussions took place with investigators from CNM on protocol design and activities, particularly on the selection of trial drugs. Following agreements on trial design and approval from CNM, an orientation meeting was held with the representatives from provincial and local health departments, district administration, village leaders, and VMWs prior to the implementation of the trial activities. During the meeting, information related to trial design and planned activities was presented in detail. Concerns and recommendations from the meeting were addressed and incorporated into trial implementation activities (Fig. 3).

2. Co-development of informational materials

**Fig. 4:**
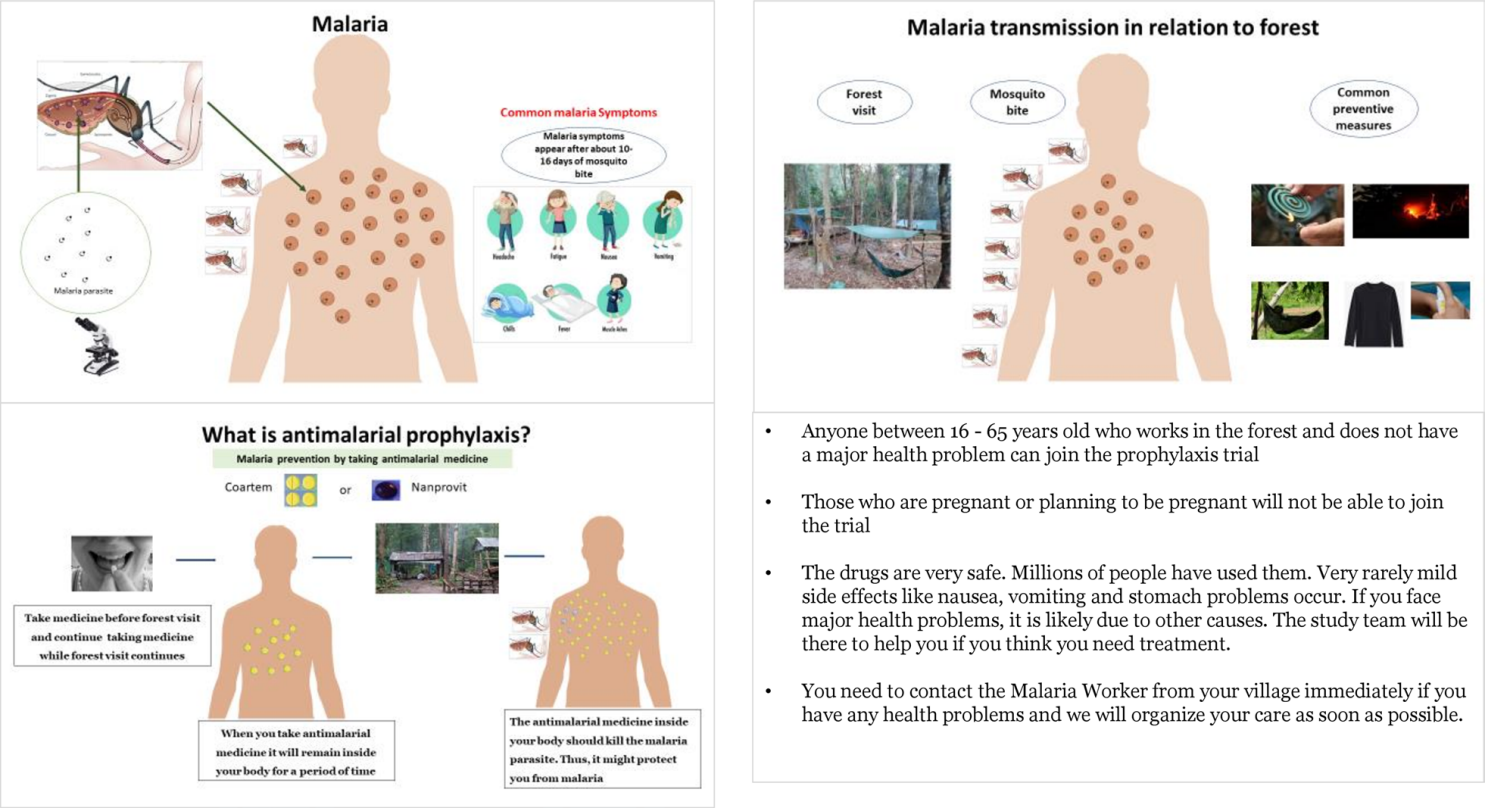
Key messages about malaria and prophylaxis

Explaining the trial rationale and its procedures to potential participants and the wider community in a comprehensible way, adapted to their language, ethnic and educational backgrounds was felt likely to be a significant challenge. Prior to engagement activities in the villages, posters that presented key messages of trial-related information were prepared jointly with stakeholders (Fig. 4). They included a brief introduction of the trial team and their activities and roles within the project. It was clarified that this was a collaborative health research project for malaria prevention implemented by CNM, the Provincial Health Department, local administration, local health authority and Mahidol Oxford Tropical Medicine Research Unit. Key messages included information on: What is malaria? How is malaria transmitted? What can happen when someone visits forested areas? What are the preventive measures commonly used? What is the proposed project about? How does chemoprophylaxis work? Who is eligible to join the trial? What do participants need to do when they join the trial? Are the trial drugs safe? What support will participants receive if they have any health problems after joining the trial? These key messages were used in meetings and discussions with community members.

3. Formation of a joint engagement team with stakeholders

A joint engagement team was formed with stakeholders during the orientation meeting to conduct engagement activities in all trial villages and to address any concerns, rumours or other challenges that emerged during the implementation of the trial activities. The stakeholder-researcher team consisted of representatives from local administration, health authority, community leaders, VMWs and trial staff. Two large meetings were held by the team prior to the recruitment of trial participants in the initial two villages in March 2020. Due to the onset of the COVID-19 pandemic, meetings in subsequent villages had to be held on a smaller scale.

VMWs and village and community leaders took the central role in organizing and conducting the meetings. They selected the venues and went house-to-house to invite the villagers to the meeting and distributed an invitation letter to each family in the village. Trial staff were introduced by the engagement team to the community members during the meetings.

4. Conducting group and individual consent

The consent process took place in several stages. Firstly, trial information was presented to all invited community members using the posters showing key messages. Next, interested groups of forest goers were invited to join the full consent process. Written informed consent was obtained from all participants prior to enrolment. For those who could not read or write, the information sheet and consent form were read to them and a fingerprint was obtained and the form countersigned by an impartial witness. A copy of the signed informed consent documents was given to the participants. For participants aged 16 to 18 years, written informed assent was obtained in addition to their parent or guardian signing a consent form.

Caution was taken to maintain privacy strictly with every female participant at fertile age to make them feel comfortable during the consent process. A female team member discussed with each female participant and her partner in private. Specifically, if they were willing to use condoms during and after the written informed consent (at the start of the study), they were enrolled.

5. Addressing the needs and concerns of the community

The study population resides in remote areas and most villages do not have convenient access to health care. Considering the situation, the engagement team undertook meetings with community health workers (village malaria workers and mobile malaria workers), and community representatives (village leaders) about the common health problems in the village. The team concluded that it would be important to offer support to the community for the health problems they face, otherwise there could be a negative impact on the acceptability of the trial. The team identified some common health problems, such as muscle pain, minor wounds, gastritis, diarrhoea, and vitamin deficiency that were of concern to the community and within the scope of the trial staff to support. Medications for these health problems were offered to the participants and villagers irrespective of their participation in the trial. In addition, to avoid pregnancy during participation in the trial, counselling was provided and offered condoms to female participants as part of provisional health care to participants.

Health check-ups such as measuring blood pressure were offered to participants in high-risk groups and those found to be hypertensive were advised to go to the health centre. The trial team were available to address all kinds of adverse events regardless of their relationship to the study drugs. Being prompt and responsive to trial participants’ complaints and offering a sense of continuous presence in the community built trust and relationships. The trial team were also proactive to meet the challenges related to participation in the trial. For instance, when participants were not able to participate in the study or were unable to come for follow-up because they were busy, the team tried to minimize these barriers by either reaching out to them with study drugs, or simply providing them with the means of transportation.

6. Selection of community representatives and allocating responsibilities

The population of the trial area consists of many ethnic minority groups, often speaking different native languages. This diversity was also observed within a single village. It was vital to involve representatives from different minority groups in project activities to ensure that they were not excluded from the research. A village leader or VMW who belonged to a particular ethnic minority was selected to take additional responsibilities e.g. explaining key trial messages, assisting with the consent process, explaining trial procedures (particularly drug administration), fielding questions and doubts from participants, and mediating discussions. Approximately 54% of VMWs were female and only one village leader was female. To balance the gender gap of representation, a female representative was selected in each village where there was no female VMW. This was done following suggestions from local community members.

7. Sharing responsibilities with trial participants

During the implementation process, previous research findings that forest goers travel to the forest in groups of up to 10 persons [3] were reconfirmed. Each group was guided by a team leader, which is usually a senior community member. Each team leader was therefore recruited as a trial volunteer, and they were briefed and offered the responsibility to guide and supervise the group for drug intake whilst they were in the forest. If not willing or capable, another group member was chosen by the group and was assigned these tasks. A simple drug calendar was developed in the local language for each participant in order to make it easier to remember the drug-dosing schedule. The selected volunteer was also responsible for countersigning drug calendars of all members of their group.

### Compensation for opportunity costs

No incentives were provided to participants during engagement meetings with community members. However, stakeholders advised that because forest workers had to travel long distances, usually on foot, and had to wait while trial staff were performing the research procedures e.g. obtaining written informed consent, data and sample collection, compensation was considered appropriate on enrolment and follow-up visits. Hence, on each of these visits, 5 US dollars were provided to each participant to compensate for loss of income. No incentive was provided to the participants to take the trial medication during forest visits, and the post forest period. Group leaders of forest goers received 50 US dollars to directly observe the self-administration of the trial medication for a maximum period of 4 months, to guide the team members for follow-up visits, and to complete and maintain the drug administration record.

### Adapting engagement activities during COVID-19

In Cambodia, COVID-19 had an important impact on the implementation of engagement activities for the trial. On 27 January 2020, the Ministry of Health (MoH) of the Kingdom of Cambodia, confirmed the detection of the first case of COVID-19 [31]. Although the activities were not affected at the beginning of 2020 because there was no SARS-CoV-2 transmission or lockdown in the trial area, there was serious concern among communities as news spread across the country. Community members were particularly worried about large gatherings and meetings inside villages. These concerns were discussed with stakeholders and village leaders and, although there were no cases in the study areas and neighbouring regions, based on their recommendations engagement activities were cancelled on the 18^th^ March 2020. It was decided to review the situation on a daily basis and follow the government guidance. Engagement activities were adapted to minimize the risk of COVID-19 transmission and restarted on the 29th April 2020. Participants were divided into smaller groups consisting of a maximum of 20 potential recruits. Some meetings were held at the research centre instead of villages and precautionary measures e.g. use of facemasks, disinfection, and appropriate physical distancing were implemented. Those who had flu-like symptoms were asked not to join. Village leaders and malaria workers played a key role in organizing and inviting participants to come to the research centre or a central village venue.

### Statistical methods

The engagement team and trial staff documented meeting reports and recorded field notes during all engagement activities and throughout the trial implementation. These have been summarized in this manuscript.

The effectiveness of the engagement strategies was determined through the proportion of forest goers participating at each stage of the trial: enrolment, compliance with trial drug intake and attendance at follow-up visits. The Pearson Chi-squared test and logistic regression were used to determine the factors associated with compliance and discontinuation of study drugs.

At any time point during the follow-up period, when a participant indicated that they did not plan to return to the forest within the next month, a further 28 days of trial drug was distributed as a post-forest period terminal chemoprophylaxis and the participants were regarded as having completed the trial and complied with the trial procedures. If a participant had a clinical malaria episode detected by rapid diagnostic test at any time during the follow-up period, the participant received anti-malarial treatment according to national treatment guidelines. No further follow-up was required and the participant was regarded as having completed the trial. When a participant was away from the village and did not come for further follow-up to receive the trial drug, they were considered as being lost to follow-up. When a participant indicated at any time point during follow-up that they did not want to continue the drug they were classified as having discontinued taking the drug.

### Findings

#### Feedback and recommendations during meetings with stakeholders

Several discussions took place with CNM at the initial stage to develop the trial protocol (Table 1 and Appendix 1). Major decisions were made based on the national malaria elimination strategy [32] e.g. selection of trial drugs, frequency and duration of anti-malarial drug administration, and suitable trial location. Artemether-lumefantrine (AL) as anti-malarial chemoprophylaxis and a multivitamin (MV) as comparator were selected following recommendations from CNM. During discussions and meetings with the local health department and administration, implementation strategies were finalized and key messages on trial procedures were developed (Fig. 2). Major recommendations from stakeholders were to form a joint team to implement engagement activities in villages; to illustrate the trial information through posters consisting of pictorial messages; to explain in detail the safety of the trial drugs; to clarify the possible adverse effects that may occur after taking trial drugs and their relationship to the trial drug; to provide assurance about clinical support for adverse effects; and to address any rumours or concerns that may occur during the trial implementation period.

**Table 1:** Feedback and decisions from engagement meetings

Stakeholders	Key queries, feedback and recommendations	Responses by engagement team
Policy makers e.g. National Malaria Control Program, Cambodia	• Recommendation that Siem Pang district bordering with southern Lao PDR as suitable study site based on malaria transmission and forest coverage • Choice of trial drugs: artemether-lumefantrine (AL) as antimalarial prophylaxis and multivitamin (MV) as control • Frequency and duration of antimalarial drug administration as prophylaxis	The recommendations and decisions were incorporated into the protocol design and procedures
Local health and administrative authorities e.g. Provincial and District Health Department, Local Governor Office representatives, commune and village leaders, Village Malaria Workers	• To form a joint team to conduct engagement activities with villagers and forest goers • To use the words “Health Project” instead of “Research Project” while explaining the trial to participants to avoid confusion and misunderstanding within the community • To simplify the trial information using posters and pictorial messages • To explain in detail the safety of the trial drugs, to clarify the possible side effects that may occur after taking them, to explain the side effects that may not be related but participants may associate with the trial drug, to ensure support is available for any side effects experienced by participants • To address any rumours or concerns that may occur during the trial period by the joint engagement team in the community	The feedback and recommendations were incorporated into the trial implementation and engagement activities. A joint engagement team was formed, and trial procedures were simplified through use of posters and leaflets. Drug safety was discussed with the villagers and their concerns were addressed jointly by the engagement team and trial staff
Villagers and forest goers	• Participants wondered why the yellow medicine (AL) had so many tablets (4 per dose) compared to the pink one (MV) • *Participants asked why children under 16 years are not allowed to join the trial and how they could be protected from malaria when they go to the forest with their parents* • Participants asked if the drug is the same as the one used for malaria treatment in the health centres • Forest goers wondered how long the drug remains in the body and how it works • Female participants asked if they could take the study drug while using a contraceptive drug regularly • Forest goers wanted to know if the study drug can be dangerous or poisonous • Participants wanted to know if they can continue smoking tobacco and drinking alcohol	We listed the possible queries that may arise from villagers and forest goers. Responses to the queries were prepared in advance and agreed with members of joint engagement team before approaching the villagers and the community We explained that the drugs are safe and millions of patients have already used them among all age groups. Participants who are pregnant and below 16 years were not included because the National Malaria Control Program first wanted to know whether the proposed antimalarial prophylaxis is effective, safe, and acceptable to the community and in adult participants Participants were advised to avoid drinking alcohol while taking study drugs and female participants were advised to ask their partners to use condoms to avoid any drug interactions with contraceptive medication

#### Major issues and feedback from community members and trial participants

While conducting engagement meetings in villages (Table 1 and Appendix 1), the most frequent issues raised by participants and community members were: how long the drugs stayed in the body; how the drugs worked; whether the trial anti-malarial was the same as the one used for current malaria treatment; why children under 16 years were not included (as they also occasionally accompany their parents to the forest); whether female participants could take the trial drug while using contraceptive medications; whether trial drugs were dangerous or poisonous; and whether it was safe to drink alcohol or smoke while taking the drugs.

#### Compliance with trial procedures

In total, 1,613 participants were assessed for eligibility and 1480 (92%) joined the trial (Table 2). Among enrolled participants, 1242 (84%) received the study drugs and completed the trial (AL: 82.2% versus MV: 85.6%, p = 0.08); 157 (11%) participants did not attend follow-up (AL: 11% versus MV: 11%, p = 0.79); and 73 (5%) participants did not want to continue the study drug (AL-7% versus MV-3%, p = 0.005). Eight (0.5%) pregnancies were recorded during the trial period, of which 4 were in the AL arm and 4 were in the MV arm.

**Table 2:** Compliance with trial procedures among forest goers in northeastern Cambodia

Characteristics	Total N (%)	AL n (%)	MV n (%)	p-value^*^
Enrolled	1480 (100.0)	738 (100.0)	742 (100.0)	
Completed the trial and received prophylaxis	1242 (84)	607 (82)	635 (86)	P = 0.081
Lost to follow up	157 (11)	79 (11)	78 (11)	P = 0.786
Received drugs at baseline	37/157 (24)	18/79 (23)	19/79 (24)	
Received drugs at least 1 month	54/157 (34)	25 /79 (32)	29/79 (37)	
Received drugs at least 2 months	66/157 (42)	36/79 (46)	30 (38)	
Discontinued taking drug	73 (5)	48 (7)	25 (3)	P = 0.005
Did not receive drug for a month	5/73 (7)	4/48 (8)	1/25 (4)	
Received drug for at least 1 month	58/73 (79)	35/48 (73)	23/25 (92)	
Received drug for at least 2 months	8/73 (11)	7/48 (15)	1/25 (4)	
Received drug for at least 3 months	2/73 (3)	2/48 (4)	0	
Pregnancy	8 (0.5)	4 (0.5)	4 (0.5)	

^*^P-value by Pearson Chi Square test

Among those who were lost to follow-up, 54/157 (34%) received at least one month and 66/157 (42%) received at least two months of study drug prophylaxis. Young participants were more likely to be lost to follow-up (age 30 years or below: 102/794, 13%; above 30 to 40 years: 33/359, 9% and above 40 years: 22/327, 7%; p = 0.003) (Table 3).

**Table 3:** Factors associated with compliance with trial procedures

Variables	Total enrolled N	Completed the trial and received prophylaxis n (%)	p- value	Lost to follow up n (%)	p- value	Discontinued taking drug n (%)	p-value
Treatment Arm			0.08		0.90		0.01^*^
Al	738	607 (82)		79 (11)		48 (7)	
MV	742	635 (86)		78 (11)		25 (3)	
Sex			0.56		0.002^*^		0.005^*^
Female	345	286 (83)		20 (6)		31 (9)	
Male	1135	956 (84)		137 (12)		42 (4)	
Age groups (years)			0.05		0.003^*^		0.61
Age ≤ 30	794	651 (82)		102 (13)		35 (4)	
Age > 30 and ≤ 40	359	304 (85)		33 (9)		20 (6)	
Age > 40	327	287 (88)		22 (7)		18 (5)	
Occupation			0.85		0.24		0.45
With less forest exposure	40	34 (85)		2 (5)		3 (7)	
With more forest exposure	1440	1208 (84)		155 (11)		70 (5)	
Past history of malaria			0.53		0.08		0.02^*^
No	644	536 (83)		58 (9)		45 (7)	
Yes	836	706 (84)		99 (12)		28 (3)	

^*^Adjusted p-value derived from logistic regression

Overall, refusal to continue with the study drug prophylaxis was remarkably low (5%; 73/1480). Among those who discontinued, 58 (79%) received drugs for at least one month, 8/73 (11%) for two months, and 2/73 (3%) for three months. The AL arm was associated with discontinuation of the study drug (AL: 48/738, 7% vs 25/742, 3%; p = 0.01). Females (31/345, 9%) were more likely (42/1135, 4%) to discontinue taking drugs at some point in the trial (p = 0.005). Those (45/644, 7%) who had no previous history of malaria infection were more likely to discontinue the study drug than those (28/836, 3%) who had a history of malaria (p = 0.02). Among 73 participants who discontinued taking the study drug, 40/73 (55%) were from one village. No significant differences were observed in the proportions of participants who received study drug prophylaxis and complied for the whole trial duration between villages in the intervention (AL) and control arm (multivitamin) (Table 4).

**Table 4:** Compliance with trial procedures among forest goers in fifteen villages in northeastern Cambodia

Village code	Enrolled			Complied with trial duration and received prophylaxis			Lost to follow up			Discontinued taking drug			Withdrawn due to pregnancy		
	Total N	AL n/N (%)	MV n/N (%)	Total n/N (%)	AL n/N (%)	MV n/N (%)	Total n/N (%)	AL n/N (%)	MV n /N(%)	Total n/N (%)	AL n/N (%)	MV n/N (%)	Total n/N (%)	AL (n)	MV (n)
3	54	27 (50.0)	27 (50.0)	45 (83.3)	22 (40.7)	23 (42.6)	5 (9.3)	2 (3.7)	3 (5.6)	0 (0.0)	0	0	4 (7.4)	3	1
5	150	75 (50.0)	75 (50.0)	133 (88.7)	64 (42.7)	69 46.0	10 (6.7)	6 (4.0)	4 (2.7)	5 (3.3)	4 (2.7)	1 (0.7)	2 (1.3)	1	1
8	43	22 (51.2)	21 (48.8)	32 (74.4)	17 (39.5)	15 (34.9)	11 (25.6)	5 (11.6)	6 (14.0)	0 (0.0)	0	0	0	0	0
9	113	56 (49.6)	57 (50.4)	100 (88.5)	51 (45.1)	49 (43.4)	10 (8.8)	4 (3.5)	6 (5.3)	2 (1.8)	1 (0.9)	1 (0.9)	1 (0.9)	0	1
10	86	42 (48.8)	44 (51.2)	70 (81.4)	33 (38.4)	37 (43.0)	10 (11.6)	4 (4.7)	6 (7.0)	6 (7.0)	5 (5.8)	1 (1.2)	0	0	0
12	147	73 (49.7)	74 (50.3)	99 (67.3)	47 (32.0)	52 (35.4)	8 (5.4)	4 (2.7)	4 (2.7)	40 (27.2)	22 (15.0)	18 (12.2)	0	0	0
13	91	46 (50.5)	45 (49.5)	72 (79.1)	35 (38.5)	37 (40.7)	15 (16.5)	8 (8.8)	7 (7.7)	4 (4.4)	3 (3.3)	1 (1.1)	0	0	0
14	117	59 (50.4)	58 (49.6)	105 (89.7)	53 (45.3)	52 (44.4)	7 (6.0)	2 (1.7)	5 (4.3)	4 (3.4)	4 (3.4)	0	1 (0.9)	0	1
17	59	29 (49.2)	30 (50.8)	50 (84.7)	25 (42.4)	25 (42.4)	8 (13.6)	3 (5.1)	5 (8.5)	1 (1.7)	1 (1.7)	0	0	0	0
19	110	55 (50.0)	55 (50.0)	101 (91.8)	50 (45.5)	51 (46.4)	8 (7.3)	4 (3.6)	4 (3.6)	1 (0.9)	1 (0.9)	0	0	0	0
20	148	74 (50.0)	74 (50.0)	144 (97.3)	74 (50.0)	70 (47.3)	4 (2.7)	0	4 (2.7)	0 (0.0)	0	0	0	0	0
22	40	20 (50.0)	20 (50.0)	38 (95.0)	18 (45)	20 (50)	0 (0.0)	0	0	2 (5.0)	2 (5.0)	0	0	0	0
23	98	49 (50.0)	49 (50.0)	74 (75.5)	35 (35.7)	39 (39.8)	23 (23.5)	13 (13.3)	10 (10.2)	1 (1.0)	1 (1.0)	0	0	0	0
24	149	75 (50.3)	74 (49.7)	119 (79.9)	58 (38.9)	61 (40.9)	27 (18.1)	15 (10.1)	12 (8.1)	3 (2.0)	2 (1.3)	1 (0.7)	0	0	0
25	75	36 (48.0)	39 (52.0)	60 (80.0)	25 (33.3)	35 (46.7)	11 (14.7)	9 (12.0)	2 (2.7)	4 (5.3)	2 (2.7)	2 (2.7)	0	0	0

#### Other major challenges during trial implementation

Challenges that occurred during trial implementation were documented by the engagement team and the trial staff (Table 5). From meeting reports and field notes, it was found that some participants were concerned about the large number of tablets that were to be taken in the AL arm (4 tablets per dose) compared to participants in the multivitamin arm (1 tablet per dose). Young married females were generally not interested in using contraceptives as they planned on having children imminently. Other female participants could not join the trial as their partner did not want to use condoms and thus could not confirm that they would avoid pregnancy whilst in the trial. Communities from the Kavet ethnic group tend to desire large families and are therefore not familiar with the use of any contraceptive method. This was a barrier for female participants to join the trial. In one village, the Muslim community were observing Ramadan and were required to abstain from food, drink or oral medication from dawn to dusk meaning they would be unable to comply with taking the study drug. In one village, predominately of Lao ethnic minority, trial staff observed a high proportion of trial dropouts. Several possible reasons were identified following interviews with participants in this village, VMWs, and trial staff: 1. The harvest season was finished (December), so most villagers returned from the forest and stayed at home so did not feel it necessary to continue the drugs. 2. Some participants moved away from their village to find jobs elsewhere after harvesting (in January) and were, therefore, unable to continue follow-up. 3. Rumours spread that the study drug caused frequent adverse effects e.g. stomach problems.

**Table 5:** Major challenges during trial implementation

Generic challenges	Specific challenges	Measures taken by the joint engagement team and trial staff to address the challenges
Taking prophylactic/unfamiliar medicine	Participants were concerned about taking a high number of tablets, especially in the arthemether-lumefantrine arm (4 tablets twice daily for 3 days followed by weekly twice a day), compared to the multivitamin arm (1 tablet twice daily for 3 days followed by weekly)	It was highlighted that the drug was safe, and the doses were standardized. Possible side effects were explained
Issues related to pregnancy	Although it was clarified to avoid becoming pregnant while taking part in the trial, eight participants were found to be pregnant following drug administration at the start of the trial. Some female participants could not join the trial as they or their partners did not want to use condoms and thus could not confirm avoiding pregnancy while on prophylaxis. Often couples were not interested in using contraceptives and wished for more children	After the first few cases of pregnant participants, the enrolment procedure was adapted: every female participant at fertile age would discuss with their partner and a team member in private. Specifically, if they were willing to use condoms during and after the written informed consent (at the start of the study), they were enrolled
Cultural considerations	In one village meeting, the VMW and village leader invited the two different ethnic groups (Muslims and Kavet) at different times for the engagement meeting and trial enrolment. The engagement team did not realize that Muslim communities were fasting because of Ramadan, when any kind of food and liquid including drugs were avoided from dawn to the dusk. When the trial team explained to couples from the Kavet ethnic minority about how to use condoms to avoid pregnancy, participants refused to join the trial as they did not want to use any contraceptives. The commune leader provided additional information that the Kavet ethnic groups like to maintain their tradition of having many children to expand the family (as assets) and so, they have never seen and used the condoms	Religious and cultural aspects were respected and taken into consideration during enrollment process. These aspects were discussed with the community and village heads prior to activities and enrolment procedures were adopted
Local perception of malaria as not a major health problem in the area	In one village with Lao ethnic minority, trial staff observed a high proportion of dropouts compared to other villages. The team thought because of their language, they may not have understood the study information. Another reason for high dropouts were that they were highly mobile, particularly they moved between places after the rice harvesting season (January) in search of jobs	The joint team identified possible reasons by interviewing participants, VMW, and the local trial staff. In December/January, after the completion of rice harvesting, most villagers did not go to the forest, so they did not feel the need to take the drugs. Participants and their group leaders were asked at enrolment if they would be able to attend the upcoming follow-up appointments
Messaging and adherence to drug regimens	On some occasions, trial staff did not involve representatives from all ethnic minority groups which meant some of these ethnic population did not understand the information about the study. Specifically, participants of Kavet minority group did not clearly understand the drug dosing schedule. Biscuits were supplied to the participants to take with drug as fatty food increases AL absorption but some participants gave the biscuits to their children, and they ran out of biscuits to take with the drug in the forest	The information about the trial activities were explained through easy to understand self-explanatory posters. If necessary, a volunteer, usually the local VMW was involved in the study clarification and discussion as a translator. Participants were provided additional biscuits and advised to take the drug with any other food containing fat
Operational challenges due to high mobility of forest goers	In one village, many participants were still in the forest on scheduled follow up days and trial staff could not reach them in time. People from most villages had farms (usually rice farms) in the forest where they stayed for several months during the rainy season and could not come back for the follow-up. Some of our participants had rice fields far away from the village and could not get back during the follow-up schedules. Some other participants when they got sick during their work in the farmlands, they sought VMWs/MMWs from other villages and thus were untraceable. Some members of forest goer groups were not going to the same location in the forest. Thus, at the follow-up, only few group members came, but not others. Some forest goer groups were big which made it difficult for group leaders to supervise intake of drugs among the members. Some people initially selected by the group as leaders of forest goer groups were illiterate	The trial team attempted to clarify at the enrolment stage whether participants would be able to attend the scheduled follow-up in a month's time. At the time of the planned follow-up, contact was made via the VMW to fix a day when participants were in their village. If necessary, the team visited villages and the farms for several days in a row and tried to establish contact with participants in the forest. In some cases, the study team also drove to the fields in the forest to conduct the follow-up at the place where the participants were staying. When group leaders of forest goers were illiterate, another member was chosen, with the groups’ agreement, even if he or she was younger than other group members
Rumors and adverse events	In one village, false rumors spread that drug causes frequent side effects e.g. stomach problems	All questions regarding the drug tolerance and safety were discussed and side effects were explained
Impact of COVID-19	First case of COVID-19 was confirmed by the Ministry of Health (MoH) of the Kingdom of Cambodia On 27 January 2020. There was serious concern among communities as news spread across the country. Villagers were particularly worried about large gatherings and meetings inside villages In late March/April 2020, some forest goers came back to their homes instead of staying in the forest because of COVID-19. Some village leaders did not want people from outside to come to their village	Discussions took place with all stakeholders including local administration and community leaders to adjust the engagement and trial activities. Decisions were made to observe the situation on daily basis and follow the guidance from government policy and measures. All meetings were only held after prior discussion and agreement with village authorities. In some cases, it was agreed that participants would travel to the main research facility for enrolments and follow-ups. In other cases, meetings within villages were held only in small groups, complying with the current COVID-19 safety measures. All government recommendations to minimize COVID-19 transmission were followed including mask wearing, hand washing and social distancing

## Discussion

The main objective of the study was to determine the effectiveness and feasibility of engaging forest-goers in a randomized controlled clinical trial of anti-malarial chemoprophylaxis with artemether-lumefantrine (AL) in northeast Cambodia. The community engagement strategy was built on and evolved from the feedback and recommendations from stakeholders. The engagement team, consisting of local community members and research staff, conducted meetings in the community and spent time together throughout the trial period, which strengthened the working relationships and trust between those involved [19, 33, 34]. In addition, MORU’s continuous presence prior to the current trial in the community with various studies and activities (clinical trials, and engagement activities) have strengthened the familiarity, relationship, and trust with the local population. In this trial, the engagement team included local community members in the discussions related to the study, which contributed to building trust between the trial staff and the forest goers. Notably, lucrative forest work, that is logging and hunting, is illegal in Cambodia which is a major barrier to engaging forest goers. The involvement of the local administration in engagement activities helped to increase confidence among forest goers to join the project. They also pointed out to the forest goers that the trial would address malaria, their biggest fear around forest activities [35]. Motivation to join the project was further facilitated by the simplified information presented in pictorial descriptions and posters (Fig. 3)**.** This helped engagement and trial staff to understand the project and increased their capacity to effectively communicate with participants.

Feedback from community members and participants during meetings in the community helped the trial team to adopt the implementation activities. For instance, participants were worried about possible adverse effects of the trial drugs and wanted to know more about how to get support in case health problems occurred. Provision of free health care (ancillary health care) by the research team that included health check-ups and medications for common health problems was positively received by the participants and the community. Ancillary health care has been found to play an important role in promoting participation in trials [34, 36]. In addition, the trial team had a continuous presence and were prompt in responding to the concerns which are recognized to foster the trust towards the trial team and encourage participation.

A frequent recommendation from community members was to include all age groups in the trial, particularly children who travel to the forest together with their parents. In the actual trial, only people aged 16 to 65 years were eligible to participate. Reasons for this included stakeholders from CNM stating a preference to focus on adult participants, as well as practical concerns with the complex weight-based dosing in smaller children and the low recorded incidence of malaria among those under 16 in the trial area.

Overall, high (84%) compliance with the trial procedures and drug intake was observed. This finding was consistent with findings from the qualitative study [35] conducted alongside the trial where it was found from interviews with participants that prophylaxis with artemether-lumefantrine for forest goers was acceptable under trial conditions. 11% of participants did not come for follow-up as they were away from their village. Some participants remained in the forest during the planting or harvesting period, were looking for jobs elsewhere, or had moved back to their hometown after harvesting. These reasons were consistent with the findings in an earlier trial of mass drug administration in western Cambodia [6]. Although differences were observed in proportions of participants who dropped out voluntarily between study arms, the overall numbers of participant drop-outs were low (48/738, 7% in AL vs 25/742, 3% in MV, p = 0.01). Discontinuation was more common among females, mainly due to a desire to become pregnant (Table 3). Discontinuation was also associated with perceived adverse events linked to the study drugs, lowered risk of getting malaria and plans to migrate away from the village. These reasons not to participate (or to discontinue the participation) echo with the literature around community wide mass drug administration studies conducted in the Greater Mekong Subregion.

Women were less likely to participate in the trial than men (female: 23% vs male: 77%). This was mostly due to the large majority of forest goers being male. Other reasons recorded by the engagement team in their field notes without numerical estimations included: the desire to become pregnant and, therefore, not being able to comply with the trial procedures; unfamiliarity with or reluctance to use contraceptives for a longer duration; and absence of permission to participate from their husbands. In the wider literature around sexual and reproductive health, unwillingness to use contraceptives, particularly condoms, which could potentially interfere with sexual pleasure may have contributed to reluctance to participate [37]. In Southeast Asia as elsewhere, the desire to be pregnant among married women, bearing children, and expanding the family is rooted in individual, social, and cultural identities [38–41]. Evidence synthesis in global health research in the past tended to exclude women, children, disabled and marginalized populations because they were considered ‘vulnerable’. Research ethicists have countered such rationales and have advocated for a more inclusive approach to ensure that children and pregnant women are involved in research so that the evidence generation is not biased and these special populations are not systematically excluded from research participation.

The engagement strategy in this trial entailed a continuous adaptation with the concerns and feedback from the local staff, trial participants and other stakeholders. For instance, as the trial was implemented amidst the evolving COVID-19 pandemic, critical adaptations included use of face masks, maintaining social distance, frequent use of sanitizers, and reinstating hygiene to avoid possible transmission of COVID-19. Such messages were reassuring to community members as they helped to allay the ongoing fear, and build confidence and trust towards the research team. Findings in this study corroborate the qualitative research [35] conducted alongside the trial that demonstrated the role of community awareness and rationales for the prophylaxis, together with the trust and relationship with the study team in contributing to the high acceptability of anti-malarial prophylaxis. There are implications of this study for policy makers, chiefly that this model of malaria chemoprophylaxis targeting high-risk groups, such as forest goers could be a valuable additional tool for malaria elimination in the GMS. The engagement approach employed in this study was successful and could be developed and adapted to other contexts. The present report on the implementation and challenges can inform engagement strategies in other communities if chemoprophylaxis is to be scaled-up, or if alternative drug regimens for chemoprophylaxis are to be evaluated in future. The results from this report have been disseminated to policy makers at the national level, and to regional stakeholders via the Global Fund’s Regional Artemisinin Initiative network.

## Limitations

Forest workers in this study were those who were residents of the villages where the recruitment was conducted. It was not known how many migrant workers from other provinces worked periodically in forested areas of the trial province and, therefore, they could not be recruited. The field reports contained the research team`s notes, questions and topics that had been discussed with participants. Data were not collected on reasons for forest goers to participate. Although the proportion of community participants completing the trial procedures was used as a measure of the effectiveness of engagement, the participation can be influenced by several other factors including tolerance of the drug-regimen, peer pressure and economic pressure (5 USD compensation was provided for lost income). MORU’s various studies and activities such as clinical trials, epidemiological studies and engagement activities have been conducted since 2018 which may have demonstrated the continuous presence, built community relationship and the trust ultimately affecting the participation in the current trial.

There is also a need for malaria chemoprophylaxis that can be taken by women of reproductive age. Moreover, it is important that this vulnerable population should not be excluded from research, therefore, a strategy was designed for their participation in the trial. Due to the limited duration of chemoprophylaxis (3 months) a strategy was evaluated that this population can access without sacrificing childbearing.

Assessing full comprehensibility of the trial procedures and of the informed consent (study procedures, benefits and harms of study drugs) was beyond the scope of this study. Nonetheless, participants` understanding of the trial was assessed by quantifying successful participation, and by evaluating observational meeting notes, which indicated a fair amount of comprehension. These claims are based on our efforts and focused on simplifying the study contents, and promoting independent decision-making by participants.

## Conclusions

Building trust and working relationships were crucial for the implementation of a complex trial design that assigned participants responsibility for correct adherence to drug regimens, communication with the trial team, as well as in maintaining long-term collaboration with communities and marginalized groups in this malaria endemic area. In order to build trust, a carefully developed, continually adapted and long-term community engagement strategy was essential. Being flexible and responsive to the needs and concerns of the community members (e.g. ancillary health care) were critical aspects of the community engagement strategy. Each village was unique in terms of social and cultural context, for instance, ethnic backgrounds, authority structures and dynamics with the population, literacy and health conditions such that the engagement needed tailored approaches. The participatory engagement strategy required extensive planning and multiple adaptations that ultimately contributed to the successful implementation of a clinical trial in a hard-to-reach and remote population.

## Data Availability

De-identified, individual participant data that underlie this Article, along with a data dictionary describing variables in the dataset, are available to researchers whose proposed purpose of use is approved by the Mahidol Oxford Tropical Medicine Research Unit data access committee. To request the dataset, please send a signed data request form to datasharing@tropmedres.ac. The data request form can be found on online.

## Appendix 1

Summary of engagement events

**Table:**

Meeting	Place	Participants	Topics	Timeline
Initial meeting	The National Center for Parasitology Entomology and Malaria Control, Cambodia (CNM)	Investigators from CNM and MORU	• Protocol design e.g., target groups, site selection, study drugs	• Before the submission to the regulatory authority
Trial orientation meeting	Local Governor office	VMW, village leaders, commune leaders, representatives from Non-governmental organizations (NGOs), local and Provincial Health Department, CNM, Local Governor office	• A meeting was organized before the study began • VMWs, village leaders, commune leaders, NGOs and Health Center staff were invited to the meeting • Representatives from CNM, Provincial Health Department, and Local Governor office joined to facilitate the meeting • Malaria and National Elimination Strategy, study protocol, implementation procedures and role of partners were discussed in the meeting • Feedback from VMWS, village and commune leaders were sought and their advice was integrated in the implementation activities	• Before the beginning of trial
Community meeting	Village	Villagers, community leader and Village leader	• To reach out every households in the target areas, a meeting were planned in each village • Villagers particularly all household heads and adults in the village were invited to the meeting • Trial purpose, activities and knowledge on malaria were carefully delivered to the participants • Feedback and advice were sought from participants. All concerns were taken into account and recommendations were integrated in the implementations activities • Household head were asked to share the meeting information’s to the family members particularly forest goers	• In each village prior recruitment day
Forest goer meeting	Village	Forest goers, VMW, group leaders, village leaders	• To reach out directly to the trial participants, a meeting were held in each village following census • All forest goers were invited • Trial purpose, activities and knowledge on malaria were carefully delivered to the participants • Feedback and advice were sought from participants. All concerns were taken into account and recommendations were integrated in the implementations activities	• In each village during recruitment
Study close out	Health Center	VMW, community leader, NGOs, Health Center staff and PHD, CNM	• Feedback of results to the community	• At the end of trial-

## References

[1] WHO (2020). World malaria report 2020.

[2] Nofal SD, Peto TJ, Adhikari B, Tripura R, Callery J, Bui TM (2019). How can interventions that target forest-goers be tailored to accelerate malaria elimination in the Greater Mekong Subregion? A systematic review of the qualitative literature. Malar J.

[3] Hoyer S, Nguon S, Kim S, Habib N, Khim N, Sum S (2012). Focused Screening and Treatment (FSAT): a PCR-based strategy to detect malaria parasite carriers and contain drug resistant *P. falciparum*, Pailin, Cambodia. PLoS ONE.

[4] Hustedt J, Canavati SE, Rang C, Ashton RA, Khim N, Berne L (2016). Reactive case-detection of malaria in Pailin Province, Western Cambodia: lessons from a year-long evaluation in a pre-elimination setting. Malar J.

[5] von Seidlein L (2014). The failure of screening and treating as a malaria elimination strategy. PLoS Med.

[6] Tripura R, Peto TJ, Chea N, Chan D, Mukaka M, Sirithiranont P (2018). A controlled trial of mass drug administration to interrupt transmission of multidrug-resistant falciparum malaria in Cambodian villages. Clin Infect Dis.

[7] Song J, Socheat D, Tan B, Dara P, Deng C, Sokunthea S (2010). Rapid and effective malaria control in Cambodia through mass administration of artemisinin-piperaquine. Malar J.

[8] von Seidlein L, Peto TJ, Landier J, Nguyen TN, Tripura R, Phommasone K (2019). The impact of targeted malaria elimination with mass drug administrations on falciparum malaria in Southeast Asia: a cluster randomised trial. PLoS Med.

[9] Kaehler N, Adhikari B, Cheah PY, Day NPJ, Paris DH, Tanner M (2018). The promise, problems and pitfalls of mass drug administration for malaria elimination: a qualitative study with scientists and policymakers. Int Health.

[10] Bannister-Tyrrell M, Gryseels C, Sokha S, Dara L, Sereiboth N, James N (2019). Forest goers and multidrug-resistant malaria in Cambodia: an ethnographic study. Am J Trop Med Hyg.

[11] Lek D, Callery JJ, Nguon C, Debackere M, Sovannaroth S, Tripura R (2020). Tools to accelerate falciparum malaria elimination in Cambodia: a meeting report. Malar J.

[12] Vantaux A, Riehle MM, Piv E, Farley EJ, Chy S, Kim S (2021). *Anopheles* ecology, genetics and malaria transmission in northern Cambodia. Sci Rep.

[13] von Seidlein L, Peto TJ, Tripura R, Pell C, Yeung S, Kindermans JM (2019). Novel approaches to control malaria in forested areas of Southeast Asia. Trends Parasitol.

[14] Sanann N, Peto TJ, Tripura R, Callery JJ, Nguon C, Bui TM (2019). Forest work and its implications for malaria elimination: a qualitative study. Malar J.

[15] Asian Development Bank. Indigenous peoples/ethnic minorities and poverty reduction: Cambodia. 2002. https://www.adb.org/publications/indigenous-peoples-ethnic-minorities-and-poverty-reduction-cambodia. Accessed 31 Mar 2021.

[16] Adhikari B, Pell C, Phommasone K, Soundala X, Kommarasy P, Pongvongsa T (2017). Elements of effective community engagement: lessons from a targeted malaria elimination study in Lao PDR (Laos). Glob Health Action.

[17] Adhikari B, Pell C, Cheah PY (2020). Community engagement and ethical global health research. Glob Bioeth.

[18] Proctor E, Silmere H, Raghavan R, Hovmand P, Aarons G, Bunger A (2011). Outcomes for implementation research: conceptual distinctions, measurement challenges, and research agenda. Adm Policy Ment Health.

[19] Pell CL, Adhikari B, Myo Thwin M, Kajeechiwa L, Nosten S, Nosten FH (2019). Community engagement, social context and coverage of mass anti-malarial administration: comparative findings from multi-site research in the Greater Mekong sub-Region. PLoS ONE.

[20] Atkinson JA, Vallely A, Fitzgerald L, Whittaker M, Tanner M (2011). The architecture and effect of participation: a systematic review of community participation for communicable disease control and elimination. Implications for malaria elimination. Malar J.

[21] Tripura R, von Seidlein L, Sovannaroth S, Peto TJ, Callery JJ, Sokha M (2023). Antimalarial chemoprophylaxis for forest goers in Southeast Asia: an open-label, individually randomised controlled trial. Lancet Infect Dis.

[22] National Institute of Statistics. General population census of the Kingdom of Cambodia 2019. Phnom Penh, Cambodia, 2019.

[23] National Center for Parasitology, Entomology and Malaria Control. Malaria information system. Phnom Penh, Cambodia, 2021. https://mis.cnm.gov.kh/. Accessed 1 Mar 2020.

[24] Callery JJ, Sanann N, Tripura R, Buntau T, Peto TJ, Kunthea P (2020). Engaging ethnic minority communities through performance and arts: health education in Cambodian forest villages. Int Health.

[25] Maude RJ, Tripura R, Ean M, Sokha M, Peto TJ, Callery JJ (2021). Study protocol: an open-label individually randomised controlled trial to assess the efficacy of artemether-lumefantrine prophylaxis for malaria among forest goers in Cambodia. BMJ Open.

[26] Adhikari B, Phommasone K, Pongvongsa T, Soundala X, Koummarasy P, Henriques G (2018). Perceptions of asymptomatic malaria infection and their implications for malaria control and elimination in Laos. PLoS ONE.

[27] Peto TJ, Tripura R, Davoeung C, Nguon C, Nou S, Heng C (2018). Reflections on a community engagement strategy for mass antimalarial drug administration in Cambodia. Am J Trop Med Hyg.

[28] Peto TJ, Tripura R, Sanann N, Adhikari B, Callery J, Droogleever M (2018). The feasibility and acceptability of mass drug administration for malaria in Cambodia: a mixed-methods study. Trans R Soc Trop Med Hyg.

[29] O'Neill S, Dierickx S, Okebe J, Dabira E, Gryseels C, d'Alessandro U (2016). The importance of blood is infinite: conceptions of blood as life force, rumours and fear of trial participation in a Fulani Village in Rural Gambia. PLoS ONE.

[30] Pell C, Tripura R, Nguon C, Cheah P, Davoeung C, Heng C (2017). Mass anti-malarial administration in western Cambodia: a qualitative study of factors affecting coverage. Malar J.

[31] Ministry of Health responds to first positive case of new coronavirus. 2020. https://www.who.int/cambodia/news/detail/28-01-2020-ministry-of-health-responds-to-first-positive-case-of-new-coronavirus. Accessed 31 Mar 2021.

[32] National Center for Parasitology, Entomology and Malaria Control. Cambodia malaria elimination action framework 2021–2025. Phnom Penh, Cambodia, 2021.

[33] Sahan K, Pell C, Smithuis F, Phyo AK, Maung SM, Indrasuta C (2017). Community engagement and the social context of targeted malaria treatment: a qualitative study in Kayin (Karen) State, Myanmar. Malar J.

[34] Vincent R, Adhikari B, Duddy C, Richardson E, Wong G, Lavery J (2022). 'Working relationships’ across difference - a realist review of community engagement with malaria research. Wellcome Open Res.

[35] Jongdeepaisal M, Ean M, Heng C, Buntau T, Tripura R, Callery JJ (2021). Acceptability and feasibility of malaria prophylaxis for forest goers: findings from a qualitative study in Cambodia. Malar J.

[36] Adhikari B, Phommasone K, Kommarasy P, Soundala X, Souvanthong P, Pongvongsa T (2018). Why do people participate in mass anti-malarial administration? Findings from a qualitative study in Nong District, Savannakhet Province, Lao PDR (Laos). Malar J.

[37] Randolph ME, Pinkerton SD, Bogart LM, Cecil H, Abramson PR (2007). Sexual pleasure and condom use. Arch Sex Behav.

[38] Honkavuo L (2021). Women's experiences of cultural and traditional health beliefs about pregnancy and childbirth in Zambia: an ethnographic study. Health Care Women Int.

[39] Matovu JK, Makumbi F, Wanyenze RK, Serwadda D (2017). Determinants of fertility desire among married or cohabiting individuals in Rakai, Uganda: a cross-sectional study. Reprod Health.

[40] Dube L (1997). Women and kinship: comparative perspectives on gender in South and South-East Asia.

[41] Ireland MS (1993). Reconceiving women: separating motherhood from female identity.

